# Isolation, Characterization, and In Vivo Evaluation Efficacy of Lytic Bacteriophage SEP1 Against *Salmonella* Paratyphi C

**DOI:** 10.3390/v18070751

**Published:** 2026-07-07

**Authors:** Zhiyi Ge, Di Lian, Wei Zhao, Weiru Song, Shengyi Han, Chunyan Xu

**Affiliations:** 1 College of Animal Science and Technology, Tarim University, Alar 843300, China; gzy20220620@163.com (Z.G.);; 2 Engineering Laboratory of Tarim Animal Diseases Diagnosis and Control, Xinjiang Production & Construction Corps, Alar 843300, China; 3 College of Pulmonary and Critical Care Medicine, Chinese PLA General Hospital, Beijing 100853, China; 4 Center for Animal Disease Control and Prevention, Yushu 815000, China; 5 Academy of Animal Sciences and Veterinary Medicine, Qinghai University, Xining 810016, China; 6 College of Veterinary Medicine, Henan Agricultural University, Zhengzhou 450046, China

**Keywords:** *Salmonella* Paratyphi, bacteriophage, biological characteristics, genomics, phage therapy

## Abstract

Multidrug-resistant *Salmonella* Paratyphi poses a severe threat to public health. As conventional antibiotics lose efficacy against emerging resistant strains, the need to develop alternative antimicrobial agents has become increasingly urgent. In this study, we isolated and characterized a novel lytic bacteriophage, designated SEP1, from poultry sewage using the *S.* Paratyphi C strain QH as the host and systematically evaluated its therapeutic potential in a murine infection model Genomic analysis confirmed that SEP1 belongs to the genus *Felixounavirus*, with an 85,703-bp genome devoid of lysogeny-associated or virulence genes. SEP1 exhibits robust environmental stability, maintaining infectivity at 10–50 °C and pH 4–9; it has a 30 min latent period and a burst size of 133 PFU per infected cell. In vivo, SEP1 treatment conferred 100% survival in infected mice, reduced organ bacterial loads, alleviated tissue damage, and normalized inflammatory cytokine profiles. Collectively, these results demonstrate that SEP1 is a promising candidate for phage therapy targeting *S.* Paratyphi C infections.

## 1. Introduction

*Salmonella* is a facultative anaerobic Gram-negative bacterium of the family *Enterobacteriaceae*. Its remarkable adaptability enables persistent colonization in diverse agricultural environments, including soil, water, and animal feed, with the fecal–oral route serving as the primary mode of transmission [1]. The genus is clinically divided into typhoidal and nontyphoidal serovars; while nontyphoidal serovars are major global foodborne pathogens affecting both humans and animals, typhoidal serovars, including *S.* Paratyphi, are human-restricted pathogens that can cause enteric fever and pose a severe threat to public health [2]. Among these serovars contributing to the global burden of salmonellosis, *Salmonella* Paratyphi is particularly notable for causing enteric fever, a systemic illness that is much more severe than the typically self-limiting gastroenteritis associated with most foodborne serovars [3]. Humans and domestic animals serve as key reservoirs for this pathogen. Clinical outcomes of infection range from asymptomatic carriage to life-threatening septicemia and are largely shaped by host immunity and an array of sophisticated bacterial virulence mechanisms [4,5]. Although antibiotics remain the standard treatment for invasive salmonellosis, the increasing prevalence of multidrug-resistant strains and the emergence of persistent bacterial subpopulations have progressively compromised their efficacy [6,7]. Epidemiological surveys indicate that over 70% of Salmonella isolates collected from commercial livestock farms exhibit multidrug resistance. The transmission of drug-resistant Salmonella through the food chain damages livestock production and continuously escalates public health risks, highlighting an urgent need to develop alternative antimicrobial strategies for animal husbandry [8,9]. Against this backdrop, bacteriophages (phages)—viruses that specifically infect and replicate inside bacterial cells—have received renewed attention as promising antibacterial alternatives.

Phages replicate via two distinct life cycles: the lytic cycle, in which progeny virions are released by lysing host bacteria to kill pathogens rapidly, and the lysogenic cycle, in which phage genomes integrate into the host chromosome and facilitate horizontal gene transfer. Strictly lytic phages possess unique advantages for clinical and agricultural applications, including narrow host specificity, in situ self-amplification at infection sites, and negligible cytotoxicity to eukaryotic cells [10,11]. To date, *Salmonella*-targeting phages have shown broad application potential, spanning rapid pathogen detection, food safety biocontrol, and therapeutic intervention for active infections [12,13,14,15,16]. In food-producing animals, lytic phage-based strategies have been extensively investigated as a viable alternative to antibiotics. Accumulating evidence confirms that the oral administration of lytic phages effectively reduces *Salmonella* colonization in broiler chickens and pigs, mitigates foodborne transmission risk along the supply chain, and supports their practical utility as feed additives or environmental biocontrol agents within the One Health framework [17,18,19,20].

Despite this progress, the translation of phage therapy from bench-scale laboratory studies to practical livestock field applications faces well-documented challenges, including the rapid emergence of phage-resistant bacterial mutants, the demand for standardized high-titer formulations, and variable phage stability under on-farm environmental conditions [9]. More critically, the majority of existing *Salmonella* phage research has focused on globally prevalent serovars such as *S.* Enteritidis and *S.* Typhimurium. By contrast, well-characterized lytic phages targeting *S.* Paratyphi C remain extremely scarce, and systematic evaluations of the in vivo therapeutic efficacy of phage therapy for S. Paratyphi C infection remain largely unreported [21]. To address this critical research gap, we present a comprehensive characterization of SEP1, a novel lytic phage targeting *S.* Paratyphi C, detail its complete genome sequence, perform morphological and biological analyses, and further evaluate its in vivo therapeutic potential in a murine infection model. Our results demonstrate that SEP1 treatment improved survival, reduced organ bacterial burden, and alleviated clinical symptoms in infected mice under the experimental conditions, supporting its potential as a candidate for anti-*Salmonella* phage therapy.

## 2. Materials and Methods

### 2.1. Strains, Samples, and Experimental Animals

A total of 32 *Salmonella* strains, including the strain used for in vivo challenge experiments, were provided by the Engineering Laboratory of Tarim Animal Diseases Diagnosis and Control, Xinjiang Production & Construction Corps. All isolates were confirmed to be *Salmonella enterica* via 16S rRNA gene sequencing combined with standard biochemical identification. The strain panel comprised 31 clinical isolates recovered from diseased chickens, ducks, and cattle, along with 1 reference-type strain (CVCC541) obtained from the China Veterinary Culture Collection Center. Only the host strain QH was further serotyped as *S*. *enterica* serovar Paratyphi C. Sewage samples were collected from multiple poultry farms in Lanzhou, China, with sampling sites spanning a geographic range of 102°36′–104°35′ E and 35°34′–37°00′ N.

Female specific-pathogen-free (SPF) BALB/c mice (6–8 weeks of age; 16–20 g body weight) were purchased from Beijing Vital River Laboratory Animal Technology Co., Ltd. (Beijing, China). All animals were maintained under standard laboratory conditions, with ad libitum access to sterile drinking water and a commercial rodent diet (Beijing Ke’ao Xieli Feed Co., Ltd., Beijing, China). Animal experiments were conducted following a 2-day acclimatization period in strict accordance with the guidelines approved by the Science and Technology Ethics Committee of Qinghai University (Approval No. PJ202501–232) and the national standard Guidelines for the Ethical Review of Laboratory Animal Welfare (GB/T 35892–2018) [22].

### 2.2. Phage Isolation, Purification, and Morphological Observation

Phages were isolated from sludge-bearing sewage samples collected from poultry farms, with the *S.* Paratyphi C strain QH serving as the propagation host. The raw sewage samples contained insoluble organic sludge precipitates; therefore, approximately 20 g of a crude solid–liquid sewage matrix was suspended in 100 mL of SM buffer (0.01% gelatin (*w*/*v*), 100 mM of NaCl, 10 mM of MgSO_4_, and 50 mM of Tris-HCl, pH 7.5). The suspension was incubated overnight at 4 °C to fully elute phage particles from the solid sludge fraction and then centrifuged at 8000× *g* for 10 min to pellet the solid debris. The resulting aqueous supernatant was collected and filtered through a 0.22 μm pore size membrane. Subsequently, 10 mL of the filtrate was mixed with an equal volume of mid-logarithmic *S.* Paratyphi C QH culture (OD_600_ ≈ 0.6) in LB broth, incubated with shaking (180 r/min) at 37 °C for 12 h, and centrifuged (8000× *g*, 10 min). Next, 100 µL of the resulting cell-free liquid supernatant was mixed with 100 µL of fresh host culture (OD_600_ ≈ 1.2) and incubated at 37 °C for 10 min to allow phage adsorption.

Plaque isolation was performed via the double-layer agar method: A total of 200 µL of the adsorption mixture was mixed with 0.7% (*w*/*v*) Luria–Bertani (LB) soft agar and overlaid onto solid LB agar plates. Following incubation at 37 °C for 12–24 h, distinct plaques were chosen and subjected to five successive rounds of purification to obtain a homogeneous phage stock. Phage concentration and further purification were performed as described by Gavrić and Knežević [23], using CsCl density gradient centrifugation (densities: 1.45, 1.50, and 1.70 g/mL) followed by ultracentrifugation at 25,000× *g* for 4 h at 4 °C. Endotoxins were removed from the purified phage suspension using a commercial endotoxin removal kit to satisfy safety requirements for in vivo administration.

A purified phage suspension (10^9^–10^10^ PFU/mL) was applied to a 400-mesh carbon-coated copper grid and left to adsorb for 10 min. After blotting off excess liquid, the grid was negatively stained with 1% (*w*/*v*) phosphotungstic acid for 2 min. Following air-drying, the samples were examined and imaged with an HT7700 transmission electron microscope (TEM; Hitachi Ltd., Tokyo, Japan) at an accelerating voltage of 78 kV. In total, 50 phage particles were observed, and 20 morphologically intact particles were selected for size measurement and morphological characterization.

### 2.3. Lytic Spectrum, Optimal Multiplicity of Infection (MOI), and Growth Characteristics

Three sequential in vitro phenotypic assays were performed to characterize the core infective properties of the phage SEP1, including lytic host range screening, optimal propagation multiplicity of infection (MOI) determination, and lytic replication kinetic analysis.

First, the lytic spectrum of the purified phage SEP1 was determined against a panel of 32 *Salmonella* strains via the standard spot plaque assay. Briefly, 100 μL of mid-logarithmic bacterial culture (OD_600_ ≈ 0.6) was thoroughly mixed with 3 mL of 0.7% (*w*/*v*) LB soft agar and overlaid onto solid LB agar plates. It was then allowed to solidify at room temperature to form a uniform bacterial lawn. Subsequently, 3–5 μL of phage suspension was spotted onto the bacterial lawn, and the plates were kept at room temperature until the phage droplets were completely absorbed. After incubation at 37 °C for 12–24 h, plaque formation was observed, and lytic activity was graded into five categories based on plaque clarity and the extent of bacterial clearance within the spotted region.

Optimal multiplicity of infection (MOI) was assayed to identify the phage–bacterium input ratio that maximized phage propagation yield. Serially diluted phage suspensions were mixed with equal volumes of logarithmic-phase bacterial cultures to achieve MOIs of 0.0001 to 10 (each gradient was tested independently). Following a 10 min adsorption period at 37 °C, the mixtures were centrifuged (8000× *g*, 10 min) to remove unadsorbed free phage particles in the supernatant. The bacterial pellets were resuspended in 10 mL of fresh LB broth and incubated with shaking at 180 rpm and 37 °C for 5 h. Post-incubation cultures were centrifuged again, and the supernatants were filtered through a 0.22 μm membrane to remove intact bacterial cells. Phage titers were quantified via the double-layer agar method. All experiments were conducted in triplicate.

One-step growth analysis was performed using the standardized sodium azide-mediated synchronized infection method described by Necel et al. (2020) [24], with minor modifications to adapt it for *S.* Paratyphi C QH. Briefly, early exponential-phase cultures (2 × 10^8^ CFU/mL, OD_600_ ≈ 0.2) were centrifuged and resuspended in 1 mL of LB supplemented with 3 mM of sodium azide. They were then infected with the phage SEP1 at the optimal MOI of 0.01 and incubated at 37 °C for 10 min to allow for adsorption and genome injection. Unadsorbed phages were removed by three consecutive washes with 3 mM of sodium azide in LB, and the final pellet was resuspended and diluted 1:1000 into prewarmed phage-free LB (defined as time 0 of the growth curve). Infective centers were quantified at 1 min after dilution, and 100 µL samples were collected at 5 min intervals for the first 60 min and at 10 min intervals thereafter for phage titration using the double-layer agar method. Burst size was defined as the number of progeny phages released per infected bacterial cell and calculated as the ratio of the phage titer at the first lysis plateau to the initial infective center count. The initial infective center count was derived from the starting bacterial concentration multiplied by the MOI of 0.01. All experiments were performed in triplicate.

### 2.4. Environmental Stability Assays

Thermal and pH stability assays were designed and conducted to evaluate the environmental tolerance of SEP1, providing experimental evidence to support its potential practical application in agricultural biocontrol and in vivo delivery. For thermal stability, phage suspensions were incubated at 10–70 °C in 10 °C increments, with each temperature tested as an independent treatment for 1 h, followed by quantification of viable phage particles. For pH stability assays, SM buffer (initial pH of 7.5) was adjusted to target pH gradients of 2–13 with 1 M HCl (for pH 2–6) or 1 M NaOH (for pH 10–13). All pH values were verified using a calibrated laboratory pH meter immediately before use. Subsequently, 100 µL of high-titer SEP1 stock suspension (11 × 10^9^ PFU/mL) was mixed with 900 µL of pH-adjusted SM buffer (10-fold final dilution; final phage concentration of 1 × 10^8^ PFU/mL) and incubated statically at 37 °C for 1 h prior to titer enumeration. All assays were performed in triplicate.

### 2.5. Phage Genome Sequencing and Bioinformatics Analysis

The genomic DNA of the purified phage was extracted using the Bacteriophage DNA Isolation Kit (Norgen Biotek Corp., Thorold, ON, Canada). The complete genome sequence of the isolated phage has been deposited in the GenBank database under the accession number MW311372.1. Sequencing libraries were prepared using the Illumina TruSeq Nano DNA LT Library Prep Kit (Illumina, San Diego, CA, USA). Raw sequencing reads were quality-processed with Trimmomatic (v0.39) (http://www.usadellab.org/cms/?page=trimmomatic) (accessed on 23 July 2019) and assembled de novo using SPAdes (v3.12.0) (http://bioinf.spbau.ru/spades) (accessed on 26 July 2019). The physical termini and initial nucleotide position of the assembled genome were determined using PhageTerm v2.0 (https://sourceforge.net/projects/phageterm/) (accessed on 1 June 2026). Open reading frames (ORFs) were predicted and functionally annotated via BLASTp (v2.9.0) alignment against the NCBI non-redundant protein database (https://blast.ncbi.nlm.nih.gov/Blast.cgi) (accessed on 10 August 2019). Antibiotic resistance and virulence genes were screened against the ResFinder (https://cge.food.dtu.dk/services/ResFinder/) (accessed on 12 August 2019) and VirulenceFinder (https://cge.food.dtu.dk/services/VirulenceFinder/) (accessed on 12 August 2019) databases. All predicted ORFs were further screened for lysogeny-related genes (integrases, excisionases, phage repressors, transposases, lysogenic conversion genes) via BLASTp against the NCBI non-redundant protein database. Transfer RNA (tRNA) genes were predicted with tRNAscan-SE 2.0 (http://lowelab.ucsc.edu/tRNAscan-SE/) (accessed on 26 April 2026). A neighbor-joining phylogenetic tree was constructed using MEGA 7.0 [25] based on the amino acid sequences of the major capsid protein. Intergenomic similarity analysis was performed with VIRIDIC [26], and genomic synteny comparisons were visualized using Easyfig 2.2.5 [27].

### 2.6. In Vivo Experimental Design and Treatment

#### 2.6.1. Preparation of the Challenge Solution

*S.* Paratyphi C QH was cultured in LB broth at 37 °C with shaking at 180 rpm until the late logarithmic phase was reached (5.0 × 10^8^ CFU/mL). Cells were harvested by centrifugation (8000× *g*, 10 min), resuspended in sterile PBS, and adjusted to a final concentration of 2.0 × 10^8^ CFU/mL.

#### 2.6.2. Grouping and Processing

BALB/c mice were randomly allocated into two independent experimental cohorts, each comprising four identical subgroups. The first cohort was designated for phenotypic monitoring (*n* = 10 per subgroup), while the second cohort was used for the assessment of organ bacterial loads, inflammatory cytokine levels, and histopathological alterations (*n* = 12 per subgroup; 3 mice per subgroup were euthanized at each time point for sample collection; for the challenge subgroup, additional samples were collected from moribund mice at a humane endpoint). The specific grouping and treatment regimens for the four subgroups are detailed below.

Challenge group: Mice received an intraperitoneal injection of 100 μL of *S.* Paratyphi C strain QH suspension (2.0 × 10^8^ CFU/mouse). At 30 min post-infection, 100 μL of sterile PBS was administered intraperitoneally, and this regimen was repeated daily for 3 consecutive days.

Phage treatment group: Mice received the same bacterial challenge as described above. At 30 min post-infection, 100 μL of purified phage SEP1 suspension (5.0 × 10^10^ PFU/mouse, MOI = 0.01) was administered intraperitoneally, followed by daily booster injections of the same dose for the next 3 consecutive days.

PBS control group: Mice received daily intraperitoneal injections of 100 μL of sterile PBS for 3 consecutive days.

Phage control group: Mice received daily intraperitoneal injections of 100 μL of phage SEP1 suspension (5.0 × 10^10^ PFU/mL) for 3 consecutive days.

### 2.7. Sample Collection and Detection Methods

#### 2.7.1. Animal Euthanasia Method

To collect tissue and blood samples at 1, 3, 5, and 7 days post-infection, the mice were humanely euthanized in strict compliance with laboratory animal welfare ethical requirements. Briefly, the mice were anesthetized via intraperitoneal injection of pentobarbital sodium at a dose of 50 mg/kg body weight. After complete unconsciousness was confirmed, verified by the absence of corneal reflex and pain response, cervical dislocation was performed to ensure rapid and painless death.

#### 2.7.2. Mouse Phenotypic Monitoring

Survival rates were recorded daily at 9:00 a.m. Body weight was measured at the same time point each day. Body condition scores (BCSs) were assessed daily using a 5-point scoring system ranging from 0 (dead) to 5 (healthy) (Table 1) [28].

#### 2.7.3. Detection of Bacterial Loads in the Liver and Spleen

At 1, 3, 5, and 7 days post-infection, liver and spleen samples were aseptically harvested from three mice per group. A 0.5 g aliquot of each tissue was homogenized in 4.5 mL of PBS. Serial dilutions of the tissue homogenates were plated on LB agar and incubated at 37 °C for 12 h, after which bacterial loads were calculated as CFU per gram of tissue.

#### 2.7.4. Histopathological Analysis

Liver, spleen, and intestine samples were fixed in 4% paraformaldehyde, dehydrated, embedded in paraffin, sectioned at 5 μm, and stained with hematoxylin and eosin (H&E). The sections were examined with an Olympus BX53 light microscope (Olympus, Tokyo, Japan). Pathology scores were assigned according to the methods of Slaoui and Fiette [29]. as follows: 0 = no detectable pathological lesions; score 1 = minimal focal inflammatory cell infiltration; score 2 = mild multifocal inflammation; score 3 = moderate inflammation with focal tissue necrosis; and score 4 = severe diffuse inflammatory infiltration and extensive tissue damage. Two blinded pathologists independently evaluated 5 randomly selected non-overlapping fields per tissue section, and the average score was used for statistical analysis.

#### 2.7.5. Peripheral Blood Inflammatory Cytokine Levels

Peripheral blood samples were collected at the corresponding time points. The concentrations of the inflammatory cytokines IL-1β, IL-6, IL-10, and IFN-γ were quantified using commercial ELISA kits in accordance with the manufacturer’s protocol.

### 2.8. Statistical Analysis

All quantitative data are presented as means ± standard deviations (SDs) from three independent replicates. Statistical analyses were performed using GraphPad Prism 9.0. One-way analysis of variance (ANOVA) followed by the least significant difference (LSD) post hoc test was applied for multiple intergroup comparisons. *p* < 0.05 was considered to indicate significant differences, and *p* < 0.01 indicated highly significant differences.

## 3. Results

### 3.1. Isolation and Microscopy

**Figure 1:**
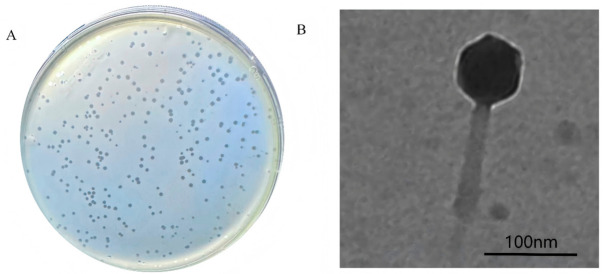
(**A**) Plaque morphology of phage SEP1 on double-layer LB agar plate (1.5% solid agar as the bottom layer, 0.7% soft agar as the overlay). (**B**) Transmission electron micrograph showing the structure of phage SEP1.

A lytic bacteriophage, designated SEP1, was isolated from poultry sewage via the double-layer agar method. Following five consecutive rounds of purification, the phage SEP1 formed clear, circular plaques surrounded by translucent halos (Figure 1A). Transmission electron microscopy (TEM) observation revealed that SEP1 possessed an icosahedral head (70 ± 1 nm in diameter) and a contractile tail (145 ± 2 nm in length) (Figure 1B).

### 3.2. Biological Characteristics

Lytic spectrum profiling demonstrated that the phage SEP1 exhibited a moderate lytic spectrum against the 32 tested *Salmonella* strains, with lytic activity detected in 10 isolates (31.25%; Table 2). No lytic activity was detected against the remaining 22 strains, which comprised 20 clinical isolates and 2 reference strains.

**Figure 2:**
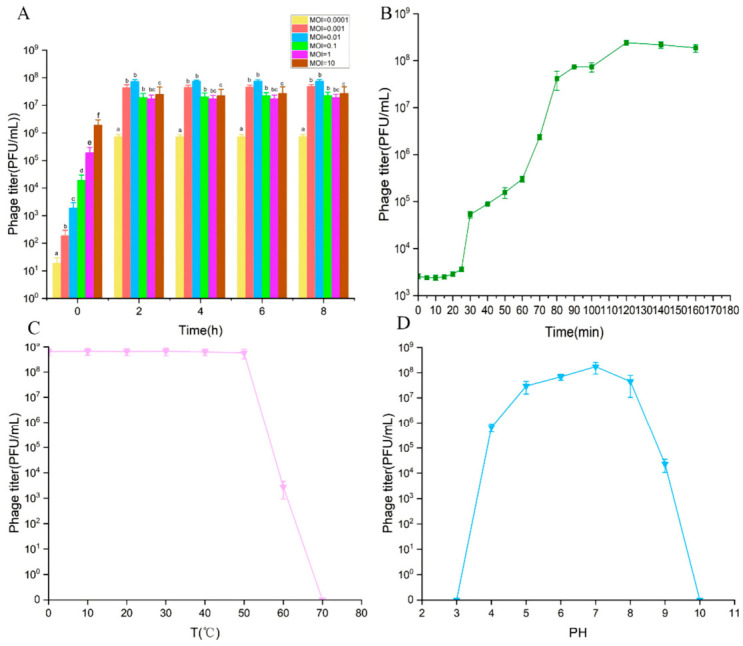
The biological characteristics of phage SEP1. (**A**) Optimal multiplicity of infection (MOI) assay. Different lowercase letters above the bars indicate statistically significant differences among MOI groups at the same time point (*p* < 0.05, one-way ANOVA followed by Tukey’s post hoc test); bars sharing a common letter are not significantly different. Bars labeled with two letters (e.g., “bc”) represent intermediate groups that are not significantly different from either the “b” or “c” groups. (**B**) One-step growth curve. (**C**) Temperature stability. (**D**) pH stability.

The optimal MOI of SEP1 was determined to be 0.01;under this condition, a maximum phage titer of 6.9 × 10^7^ PFU/mL was achieved after 4 h of propagation in *S.* Paratyphi C QH (Figure 2A).

One-step growth curve analysis was performed at an MOI of 0.01, which revealed that phage SEP1 had a latent period of 30 min, followed by an exponential proliferation phase reaching a plateau at 120 min, with a mean burst size of 133 PFU per infected cell (Figure 2B).

Thermal stability assays showed that SEP1 retained complete lytic activity between 10 °C and 50 °C, while irreversible inactivation occurred at temperatures exceeding 50 °C (Figure 2C). With respect to pH tolerance, the phage maintained infectivity across the pH range of 4–9, and complete inactivation occurred only under extremely acidic (pH < 3) or strongly alkaline (pH > 10) conditions (Figure 2D).

### 3.3. Genomic Characterization

**Figure 3:**
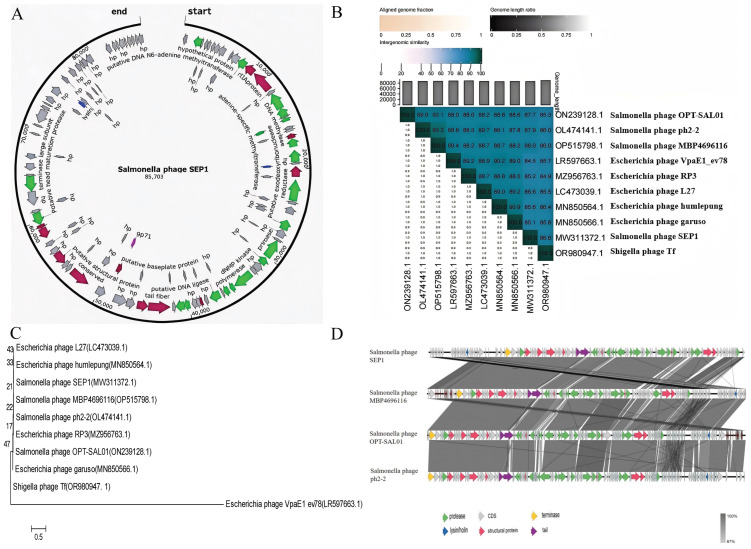
(**A**) Circular genome map of the *Salmonella* phage SEP1 (85,703 bp). The outer circle shows predicted open reading frames (ORFs), which are colored according to function: green for functional enzymes, purple for structural proteins, blue for lysin and holin proteins, and gray for hypothetical proteins. (**B**) Intergenomic similarity matrix of SEP1 and closely related phages, generated using VIRIDIC. The color scale indicates intergenomic similarity (0–100%), and the gray bars represent genome length. SEP1 shared the highest similarity (88.0%) with the *Salmonella* phage MBP4696116. (**C**) Neighbor-joining phylogenetic tree based on the major capsid protein (MCP) sequences of SEP1 and related phages. Bootstrap values (1000 replicates) are shown at the nodes. (**D**) Linear genome alignment of SEP1 with closely related *Salmonella* phages (MBP4696116, OPT-SAL01, ph2-2), generated using Easyfig. Colored arrows represent ORFs with predicted functions, and gray shading indicates regions of sequence homology (67–100% identity).

The complete genome of SEP1 (GenBank accession No. MW311372.1) is an 85,703-bp linear double-stranded DNA molecule featuring heterogeneous termini generated by a pac-dependent headful packaging mechanism. It harbors 123 predicted coding sequences (CDSs), of which 67 were functionally annotated via homology analysis, while the remaining 56 encode hypothetical proteins of uncharacterized function. Functional classification assigned these ORFs to three distinct functional modules: structural components (major capsid, portal protein, tail sheath, and tail fiber proteins), DNA replication and repair machinery (DNA helicase, primase, and polymerase), and a host lysis system (holin and endolysin). Notably, no lysogeny-associated genes (including integrases, excisionases, phage repressors, and transposases) were detected across all functionally annotated ORFs, confirming that SEP1 is a strictly lytic phage with no lysogenic conversion risk. The genome also encodes multiple tRNA genes, including high-confidence tRNAs for Ala, Arg, Cys, Gln, Gly, Leu, Pro, Thr, and Tyr. (Figure 3A, Table 3).

Phylogenetic analysis based on whole-genome similarity (Figure 3B) revealed that the *Salmonella* phage SEP1 exhibited the highest intergenomic sequence identity with several other *Salmonella* phages, including MBP4696116 (OP515798.1; 88.0% sequence identity), ph2-2 (OL474141.1; 87.9% sequence identity), and OPT-SAL01 (ON239128.1; 87.7% sequence identity). In addition to its similarity to *Salmonella* phages, SEP1 exhibited notable sequence similarity to *Escherichia* phage L27 (LC473039.1; 86.6% sequence identity), *Shigella* phage Tf (OR980947.1; 86.1% identity), and *Escherichia* phage garuso (MN850566.1; 85.1% sequence identity) [30]. All these phages belong to the genus *Felixounavirus*, indicating that SEP1 clusters within a clade of closely related members of this genus. This taxonomic assignment was further supported by a phylogenetic analysis of the amino acid sequence of the major capsid protein (MCP) (Figure 3C), which revealed that SEP1 formed a tight subclade with the *Salmonella* phages MBP4696116 and ph2-2 and that this subclade was nested within a broader clade consisting of the *Escherichia* phage garuso, *Shigella* phage Tf, and other related phages. Additionally, linear genome alignment performed with Easyfig (Figure 3D) demonstrated extensive synteny between SEP1 and closely related *Salmonella* phages (MBP4696116, OPT-SAL01, and ph2-2), with conserved functional modules encoding lysis proteins, structural components, and DNA packaging machinery. Several variable genomic regions were also identified among these phages, which likely relate to differences in their environmental adaptability and host range.

### 3.4. Clinical Manifestations of S. Paratyphi C-Infected Mice Following Phage SEP1 Treatment

**Figure 4:**
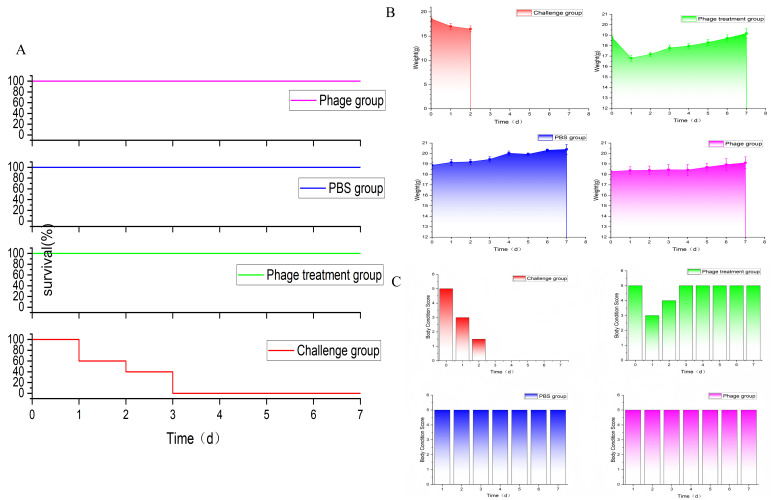
(**A**) Survival rate; (**B**) body weight changes; (**C**) body condition scoring.

All mice in the untreated challenge group succumbed to infection within 3 days. By contrast, SEP1 treatment conferred 100% survival, a level comparable to those of the PBS and phage-only control groups (Figure 4A). Untreated infected mice exhibited marked weight loss, with their body weight decreasing from 18.58 ± 0.56 g to 16.48 ± 0.62 g by day 2, alongside a rapid decline in BCS to 0 by day 3. These mice also presented with typical clinical signs of severe systemic infection, including lethargy, hunched posture, and piloerection (Figure 4B,C). In stark contrast, the phage-treated mice maintained steady weight gain, with their body weight rising from 18.80 ± 0.29 g to 19.17 ± 0.47 g over the 7-day observation window. Further, they maintained near-normal BCS, with only a transient mild decrease on day 2. Statistical analysis revealed no significant difference in body weight or BCS between the phage-treated group and the control groups (*p* > 0.05). Notably, highly significant differences were observed between the phage-treated group and the challenge group at day 2 (*p* < 0.01).

### 3.5. Bacterial Load in the Liver and Spleen of Infected Mice After Phage SEP1 Intervention

**Figure 5:**
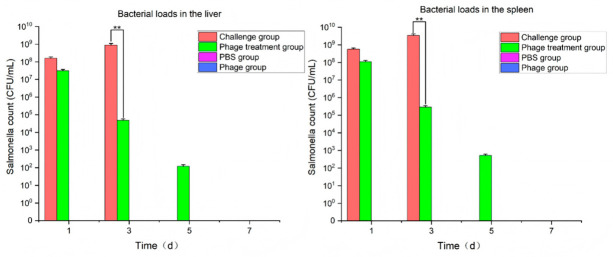
Bacterial loads (CFU/g) in the liver (**left**) and spleen (**right**) were measured at days 1, 3, 5, and 7. Data are presented as means ± SDs (*n* = 3). Statistical significance was determined by one-way ANOVA with the least significant difference (LSD) post hoc test. ** indicates a highly significant difference compared with the challenge group (p < 0.01). The challenge group mice did not survive to days 5 and 7 (ND). Phage treatment significantly reduced bacterial loads compared to the challenge group (*p* < 0.01).

The bacterial challenge induced a rapid lethal infection, with all mice in the untreated challenge group succumbing by day 3 post-infection. Accordingly, bacterial loads in this group were measured only up to day 3, revealing unchecked bacterial proliferation in both the liver and spleen (Figure 5). In contrast, phage intervention effectively controlled the infection. By day 3, bacterial loads in the treated group had already declined markedly, dropping to approximately 1 × 10^2^ CFU/g in both organs by day 5 and falling below the limit of detection by day 7. Notably, no culturable bacteria were detected in either the PBS or phage-only control groups throughout the observation period, ruling out nonspecific antibacterial effects and phage-derived confounding factors.

### 3.6. Pathological Damage to the Liver, Spleen and Gut of Infected Mice Is Alleviated by Phage SEP1

**Figure 6:**
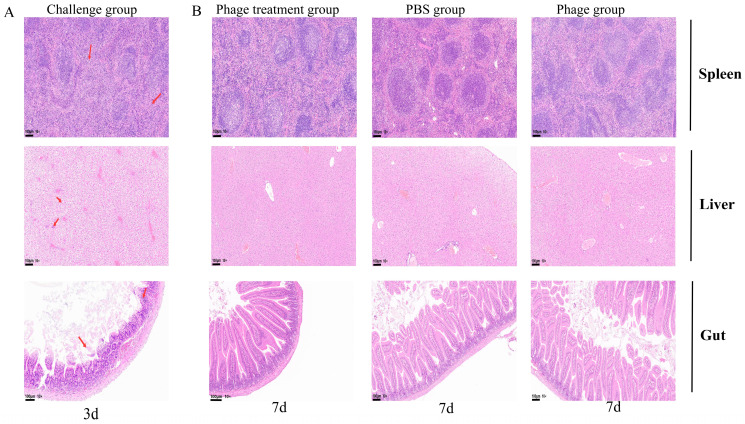
Histopathological changes in spleen, liver, and gut tissues (H&E staining). Red arrows indicate representative pathological lesions. (**A**) Challenge group at 3 days post-infection (moribund state; 100% mortality). Severe diffuse inflammatory infiltration, tissue necrosis, and structural destruction were observed in all three organs. (**B**) Phage treatment, PBS control, and phage-only groups at 7 days post-infection. Phage treatment markedly alleviated tissue injury, with only minimal inflammatory infiltration and intact tissue architecture; the two control groups showed nearly normal histology.

Tissues from the challenge group were collected at 3 days post-infection (dpi), when all mice had reached a moribund state and met the pre-defined humane endpoint criteria. Representative pathological lesions are shown in Figure 6A. By contrast, tissues from the phage-treated, PBS, and phage-only control groups were harvested at 7 dpi, when the animals remained clinically asymptomatic; the corresponding histopathological micrographs are presented in Figure 6B.

The challenge group (3 dpi) exhibited severe pathological injury across all examined organs: liver sections showed multifocal hepatocellular necrosis, inflammatory cell foci, and sinusoidal congestion (score: 3); splenic sections showed disruption of normal tissue architecture, loss of defined lymphoid follicles, and prominent inflammatory cell infiltration (score: 3); and intestinal sections presented villous atrophy, crypt epithelial necrosis, and diffuse inflammatory infiltration (score: 3).

In contrast, tissue architecture in the phage-treated group (7 dpi) remained largely preserved: the livers showed only minimal inflammation and intact lobular structure (score: 1); the spleens exhibited mild red pulp congestion and subtle follicular alterations (score: 1); and the intestines displayed largely intact villous and crypt architecture with mild edema and limited inflammation (score: 1). Minimal to mild pathological alterations (scores 0–1) were observed in both the PBS and phage-only control groups; these changes were markedly milder than those in the challenge group and comparable to those in the phage-treated group.

### 3.7. Phage SEP1 Modulates Peripheral Blood Cytokine Profiles in S. Paratyphi C-Infected Mice

**Figure 7:**
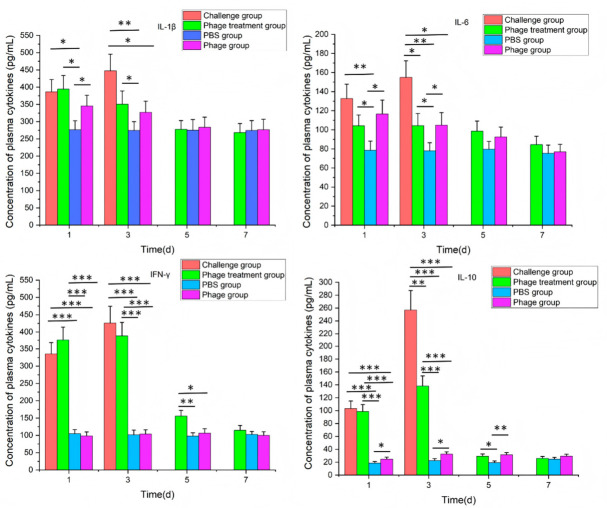
Plasma concentrations of IL-1β, IL-6, IFN-γ, and IL-10 at days 1, 3, 5, and 7 post-infection. Data are shown as mean ± SD (*n* = 3 per group). Statistical significance was determined by one-way ANOVA with the least significant difference (LSD) post hoc test for multiple comparisons at each time point. On days 1 and 3, the challenge group was compared with the PBS control, phage control, and phage-treated groups. On days 5 and 7 (no challenge group mice survived to these time points), the phage-treated group was compared with the PBS control and phage control groups. * *p* < 0.05, ** *p* < 0.01, *** *p* < 0.001. Unmarked comparisons indicate no significant difference (*p* > 0.05).

As shown in Figure 7, in the challenge group, all cytokines (IL-1β, IL-6, IL-10, and IFN-γ) were markedly elevated on day 1 and increased further by day 3; no data could be obtained beyond day 3 because all mice in this group had died. On day 1, the phage-treated group showed no statistically significant differences in any cytokines compared with the challenge group (all *p* > 0.05). On day 3, in contrast to the challenge group, the phage-treated group exhibited a significant decrease in IL-6 (*p* < 0.05) and a highly significant decrease in IL-10 (*p* < 0.01); the decrease in IL-1β was marked but not statistically significant (*p* = 0.0542), and IFN-γ levels remained unchanged (*p* > 0.05). By day 5, IL-1β and IL-6 levels in the phage-treated group were comparable to those in the PBS control group (*p* > 0.05), whereas the IL-10 level remained significantly higher than that in the control group (*p* < 0.05), and the IFN-γ level remained elevated (*p* < 0.01). By day 7, the levels of all four cytokines in the phage-treated group had normalized and were indistinguishable from those in the PBS control group (all *p* > 0.05).

## 4. Discussion

In this study, we isolated and characterized a lytic *Salmonella* phage, SEP1, and systematically evaluated its therapeutic potential in a mouse model of *S.* Paratyphi C infection. The results demonstrated that SEP1 possessed several advantageous biological traits, including stability over a temperature range of 10–50 °C and a pH range of 4–9, a short latent period, a high burst size, and a strict lytic cycle devoid of lysogeny-associated genes. These properties align with the defining phenotypic and genetic features of phages belonging to the genus *Felixounavirus*, supporting the taxonomic assignment of SEP1 to this clade [31,32]. Members of this genus are generally reported to possess a conserved genome structure and lack virulence-associated genes, further corroborating the taxonomic placement of SEP1 and underscoring its inherent biological advantages. These characteristics indicate that SEP1 holds considerable promise for biocontrol applications, as it circumvents the biosafety risks associated with temperate phages, such as the transfer of virulence factors and antibiotic resistance genes through lysogenic conversion [33,34,35]. Notably, although SEP1 is a virulent phage, this does not guarantee absolute safety; its genetic stability under prolonged environmental exposure and its potential for horizontal gene transfer with host bacteria require further investigation [36,37]. Furthermore, because whole-genome sequencing analysis relies on database comparisons, genes lacking functional annotation in NCBI databases, as well as newly emerging or potential risk-associated genes, may be overlooked [38]. Despite its moderate lytic spectrum, SEP1 lysed clinically relevant *Salmonella* isolates from diseased chickens, ducks, and cattle, highlighting its potential for targeted agricultural applications [39,40]. Nevertheless, we acknowledge that, compared with broad-spectrum antibiotics, its relatively narrow lytic spectrum may limit its direct application in clinical settings. This limitation may be overcome in the future through well-designed phage cocktail therapies, for example, by combining phages with complementary host ranges that target major *Salmonella* serotypes prevalent in clinical and agricultural settings (e.g., *Enteritidis*, *Typhimurium*, and *Infantis*), which are widely associated with animal and human salmonellosis, thereby enhancing efficacy in complex infection models [41,42]. Thus, the application of SEP1 as a monotherapy is relatively limited, and it may offer greater value as a component of rationally designed phage cocktails. Full serotyping of all lysis-positive isolates will be performed in future studies to rigorously determine the serovar-level host range of SEP1 and facilitate cocktail formulation.

In terms of in vivo therapeutic efficacy, SEP1 was shown to rescue mice from infection, highlighting its potential for clinical application. However, it should be noted that these results were obtained in a highly controlled laboratory infection model (using a single bacterial strain with a defined infection dose and timing). The treatment regimen was designed based on the pathological course of salmonellosis. The initial intraperitoneal injection, administered 30 min post-infection, ensured rapid entry of the phage into circulation to combat early bacteremia, while subsequent injections over three consecutive days aimed to maintain effective phage concentrations in the blood and target organs for sustained bacterial clearance [43,44]. Accordingly, all treated animals survived, showing only mild tissue damage and a rapid reduction in organ bacterial loads. These outcomes were associated with weight recovery, improved clinical scores, and normalization of inflammatory cytokine levels. These results were consistent with the effects reported for similar *Salmonella* phage therapies [45,46]. However, these positive indicators reflect only short-term protective efficacy within the 7-day observation window. Long-term monitoring is essential for determining the durability of phage-mediated protection, detecting potential delayed adverse effects, and assessing the risk of post-treatment infection recurrence—all of which are critical for evaluating the clinical translational value of phage therapy [47]. These endpoints were not assessed in this study but will be systematically investigated in extended in vivo experiments in future work. SEP1 treatment suppressed infection-induced cytokine storms and reduced the levels of IL-1β, IL-6, and IFN-γ, thereby contributing to the restoration of immune balance [48,49]. This immunomodulatory effect should be interpreted cautiously, as it was likely an indirect consequence of the reduced bacterial load rather than a direct immunomodulatory function of the phage. The specific mechanisms underlying this effect and its reproducibility in different immune contexts require further elucidation. Although this effect is likely indirect, these observations suggest that timely phage administration may help control excessive inflammation [50,51]. However, directly linking this immunomodulatory effect to key determinants in the progression of sepsis or bacteremia is not sufficiently supported by the existing evidence, and more refined mechanistic studies are needed to establish causal relationships.

Furthermore, the single-strain and single-phage strategy used in this study did not fully capture the genetic diversity of natural *Salmonella* populations or the complexity of polymicrobial infections [52]. *Salmonella* populations display extensive genomic variation, including differences in surface receptors that may limit the host range of a single phage—an inherent challenge for single-phage approaches [53,54]. Moreover, we did not evaluate the efficacy of SEP1 against a broader panel of clinical isolates, particularly multidrug-resistant strains, nor did we assess its effectiveness in coinfection or polymicrobial competition settings. Future studies should evaluate the therapeutic potential of SEP1 in more complex scenarios, such as polymicrobial infections and biofilm-associated models, and in other animal species.

In summary, in this study, we characterized the biological properties, genomic features, and therapeutic efficacy of the *Salmonella* phage SEP1. Given its well-characterized genome, robust environmental stability, favorable safety profile, and therapeutic efficacy, SEP1 warrants further development as a component of precision antibacterial strategies, particularly in the face of increasing antibiotic resistance. However, many obstacles to the translation of phages from preclinical research to successful clinical application remain. These include, but are not limited to, establishing scalable production and purification processes, evaluating formulation stability, conducting rigorous regulatory assessments, and performing clinical trials to demonstrate safety and efficacy in humans [55,56,57].

## 5. Conclusions

As a novel member of the genus *Felixounavirus*, the phage SEP1 displays potent lytic activity against *S.* Paratyphi C and outstanding in vivo therapeutic efficacy. Its robust environmental stability and favorable safety profile render it a promising candidate for development as a therapeutic phage. Future work will focus on optimizing delivery routes, formulating SEP1-based phage cocktails, and assessing their long-term safety to facilitate the clinical translation of this candidate.

## Figures and Tables

**Table 1:** Body condition scoring criteria.

Score	Clinical Status	Description
5	Normal	Normal activity, feeding behavior, and mental status.
4	Mildly Impaired	Ruffled fur with mild mental depression.
3	Moderately Impaired	Diarrhea, ocular discharge, and general depression.
2	Severely Impaired	Diarrhea, ocular discharge, lethargy, and unresponsiveness to external stimuli.
1	Moribund	Severe lethargy and inactivity.
0	Dead	Death.

**Table 2:** Lytic spectrum of phage SEP1 against 32 *Salmonella* enterica isolates.

Number	Strains	Isolation Source	Lytic Activity
1	*Salmonella enterica* serovar Paratyphi C QH ^1^	Diseased chickens	++++
2	*Salmonella enterica* B32M	Diseased chickens	+++
3	*Salmonella enterica* dp41	Diseased ducks	++
4	*Salmonella enterica* frp36	Diseased ducks	+
5	*Salmonella enterica* gzp3	Diseased chickens	+
6	*Salmonella enterica* fzp3	Diseased chickens	+
7	*Salmonella enterica* gzp32	Diseased chickens	+
8	*Salmonella enterica* g22	Diseased cattle	+
9	*Salmonella enterica* m25	Diseased cattle	+
10	*Salmonella enterica* f2p25	Diseased chickens	+
11	*Salmonella enterica* gzp26	Diseased chickens	−
12	*Salmonella enterica* m113	Diseased cattle	−
13	*Salmonella enterica* f8p41	Diseased ducks	−
14	*Salmonella enterica* f2p41	Diseased ducks	−
15	*Salmonella enterica* m6	Diseased cattle	−
16	*Salmonella enterica* sm95ys	Diseased cattle	−
17	*Salmonella enterica* 0167	Diseased cattle	−
18	*Salmonella enterica* m12	Diseased cattle	−
19	*Salmonella enterica* 0174	Diseased cattle	−
20	*Salmonella enterica* m34	Diseased cattle	−
21	*Salmonella enterica* gzp16	Diseased ducks	−
22	*Salmonella enterica* YS220	Diseased ducks	−
23	*Salmonella enterica* gzp58	Diseased chickens	−
24	*Salmonella enterica* fzp29	Diseased chickens	−
25	*Salmonella enterica* dp23	Diseased chickens	−
26	*Salmonella enterica* 336650	Diseased chickens	−
27	*Salmonella enterica* fzp59	Diseased chickens	−
28	*Salmonella enterica* fcp28	Diseased chickens	−
29	*Salmonella enterica* CVCC541	Reference-type strain (CVCC)	−
30	*Salmonella enterica* dp43	Diseased chickens	−
31	*Salmonella enterica* xn2407-4	Diseased chickens	−
32	*Salmonella enterica* f2p59	Diseased chickens	−

Notes for lytic activity grading: ++++ = Complete clear lysis, full lawn elimination without residual bacterial growth. +++ = Significant lysis, >90% reduction in bacterial lawn. ++ = Partial lysis, obvious transparent zones with residual colonies. + = Weak lysis, faint turbid clearing zones only. − = No observable lytic activity, intact bacterial lawn. All grading results were validated via triplicate spot plaque assay. ^1^ This strain was serotyped to the serovar level; all other test strains in this table were only identified to the species level via biochemical assays and molecular methods.

**Table 3:** Basic characteristics of bacteriophage SEP1 genome.

Phage	Genome Size (bp)	GC Percent (%)	The Number of ORFs	The Total Length of ORFs (bp)	The Average Length of ORFs (bp)	The Percentage of ORFs (%)
SEP1	85,703	39.00	123	75,408	613.07	87.99

## Data Availability

The complete genome sequence of bacteriophage SEP1 reported in this study is publicly available in the GenBank database under the accession number MW311372.1. The full sequence can be accessed via the following link: https://www.ncbi.nlm.nih.gov/nuccore/MW311372.1 (accessed on 30 November 2020). All other relevant data supporting the findings of this study are available from the corresponding authors upon reasonable request.
